# Aflatoxin B_1_ exposure and liver cirrhosis in Guatemala: a case–control study

**DOI:** 10.1136/bmjgast-2020-000380

**Published:** 2020-07-07

**Authors:** Christian S Alvarez, Elisa Hernández, Kira Escobar, Carmen I Villagrán, María F Kroker-Lobos, Alvaro Rivera-Andrade, Joshua W Smith, Patricia A Egner, Mariana Lazo, Neal D Freedman, Eliseo Guallar, Michael Dean, Barry I Graubard, John D Groopman, Manuel Ramírez-Zea, Katherine A McGlynn

**Affiliations:** 1 Division of Cancer Epidemiology and Genetics, National Cancer Institute, Rockville, Maryland, USA; 2 Centro de Investigaciones Biomédicas, Facultad de Ciencias Médicas, Universidad de San Carlos de Guatemala, Guatemala, Guatemala; 3 INCAP Research Center for the Prevention of Chronic Diseases, Institute of Nutrition of Central America and Panama, Guatemala, Guatemala; 4 Department of Environmental Health and Engineering, Bloomberg School of Public Health, Johns Hopkins University, Baltimore, Maryland, USA; 5 Department of Medicine, School of Medicine, Johns Hopkins University, Baltimore, Maryland, USA; 6 Department of Epidemiology, Bloomberg School of Public Health, Johns Hopkins University, Baltimore, Maryland, USA

**Keywords:** liver cirrhosis, chronic liver disease, epidemiology

## Abstract

**Objective:**

In Guatemala, cirrhosis is among the 10 leading causes of death, and mortality rates have increased lately. The reasons for this heavy burden of disease are not clear as the prevalence of prominent risk factors, such as hepatitis B virus, hepatitis C virus and heavy alcohol consumption, appears to be low. Aflatoxin B_1_ (AFB_1_) exposure, however, appears to be high, and thus could be associated with the high burden of cirrhosis. Whether AFB_1_ increases the risk of cirrhosis in the absence of viral infection, however, is not clear.

**Design:**

Cirrhosis cases (n=100) from two major referral hospitals in Guatemala City were compared with controls (n=200) from a cross-sectional study. Logistic regression was used to estimate the ORs and 95% CIs of cirrhosis and quintiles of AFB_1_ in crude and adjusted models. A sex-stratified analysis was also conducted.

**Results:**

The median AFB_1_ level was significantly higher among the cases (11.4 pg/mg) than controls (5.11 pg/mg). In logistic regression analyses, higher levels of AFB_1_ was associated with cirrhosis (quintile 5 vs quintile 1, OR: 11.55; 95% CI 4.05 to 32.89). No attenuation was observed with adjustment by sex, ethnicity, hepatitis B virus status, and heavy alcohol consumption. A significantly increasing trend in association was observed in both models (p trend <0.01). Additionally, the cirrhosis–AFB_1_ association was more prominent among men.

**Conclusions:**

The current study found a significant positive association between AFB_1_ exposure and cirrhosis. Mitigation of AFB_1_ exposure and a better understanding of additional risk factors may be important to reduce the burden of cirrhosis in Guatemala.

**Summary box**

**What is already known about this subject?**

- Previous studies have reported an association between aflatoxin B_1_ (AFB_1_) and cirrhosis, particularly in populations with high prevalence of chronic infection with hepatitis B virus (HBV).
- Whether AFB_1_ increases the risk of cirrhosis in the absence of viral infection, however, has not been well examined.

**What are the new findings?**

- In this case–control study, the median AFB_1_ level was significantly higher among the cases than controls.
- Furthermore, there was a significantly increasing trend in the association between AFB_1_ and cirrhosis, even after adjustment with important covariates such as sex, ethnicity, HBV status, and heavy alcohol consumption.
- In addition, the cirrhosis–AFB_1_ association was stronger among men than women.
- To our knowledge, this is the first study to report an association between AFB_1_ and cirrhosis in Guatemala, a population with low prevalence of viral chronic hepatitis and low rate of heavy alcohol consumption.

**How might it impact on clinical practice in the foreseeable future?**

- Interventions to reduce exposure to AFB_1_ as well as effort to understand the role of other risk factors for cirrhosis may be important to reduce the burden of the disease in Guatemala.

## Introduction

Aflatoxin B_1_ (AFB_1_) is a known risk factor for hepatocellular carcinoma (HCC),^1^ the dominant type of liver cancer. In Guatemala, the estimated incidence of HCC is the highest in the Western hemisphere.^2^ The major risk factors for HCC in Guatemala are not well characterised, but the prevalence of AFB_1_ exposure appears to be high.^3^ The great majority of HCCs (≥80%) develop in persons with pre-existing cirrhosis.^4^ Therefore, insights into the relationship between AFB_1_ and cirrhosis could be informative.

With over one million deaths per year, cirrhosis is the 11th most common cause of death worldwide.^5^ In combination with HCC, cirrhosis accounts for 3.5% of all deaths globally.^5^ In Guatemala, cirrhosis is among the 10 leading causes of death and accounts for an estimated 3.4% of all premature deaths.^6^ In addition, mortality rates of cirrhosis have increased with an average annual per cent change of 14.4% over the past two decades,^7^ thus representing an important public health issue in Guatemala.

Cirrhosis is a severe chronic liver disease which occurs in response to liver injury, featuring encapsulation or replacement of the damaged liver tissue by scar tissue with distortion of the hepatic vasculature and architecture.^8^ The disease is often asymptomatic until complications such as variceal bleeding, ascites and jaundice occur.^9^ In the USA, Europe and some countries in Latin America, cirrhosis is a leading indication for liver transplantation.^10 11^ HCC and cirrhosis are known to share common risk factors, including heavy alcohol consumption, hepatitis B virus (HBV), hepatitis C virus (HCV), and the related metabolic abnormalities of obesity and non-alcoholic fatty liver disease (NAFLD).^12^ It has also been reported that AFB_1_ is associated with cirrhosis among persons infected with HBV or HCV.^13–15^ Whether AFB_1_ increases the risk of cirrhosis in the absence of viral infection, however, remains unclear.

In 2017, our group reported high levels of serum AFB_1_-albumin adducts and low prevalences of HBV (0.9%) and HCV (0.5%) infections in a cross-sectional study of Guatemalan adults.^3^ In addition, our group has found that the most important source of AFB_1_ exposure in the population was consumption of tortillas, a primary staple in the Guatemalan diet.^16^ This finding was consistent with prior evidence of high AFB_1_ levels in maize samples across the country.^17^ The current study was designed to assess the association between AFB_1_ and cirrhosis in Guatemala.

## Methods

### Study population

One hundred cirrhosis cases were ascertained between February and November 2015 at two large public hospitals in Guatemala City (Hospital General San Juan de Dios and Hospital Roosevelt). The cases were outpatients recruited at the hospitals’ outpatient clinics and emergency rooms. Cirrhosis was diagnosed by abdominal ultrasonography using a quantitative scoring system, including morphological appearance of the liver surface, liver parenchymal texture, intrahepatic vascular structure and spleen size.

Controls were selected from a cross-sectional study of Guatemalan adults, aged 40 years and older, that was conducted in 2016. The cross-sectional study enrolled 461 individuals from five departments of Guatemala in order to determine the prevalence of risk factors for liver cancer. The study recruitment was based on a non-random household visit using maps of the community when available. Details of the study have been previously described.^3^ The selection of 200 controls for the current study was based on the residence of the cirrhosis cases, 83% of whom resided in the department of Guatemala or vicinity. Hence, 85% of the controls were chosen from the departments of Guatemala and Escuintla (approximately 64 kilometers from the capital city). Individuals in the cross-sectional study who reported a history of cirrhosis were not eligible to be controls in the current analysis (n=7). A flow chart of the inclusion and exclusion criteria for cases and controls is presented in online supplementary figure S1.

10.1136/bmjgast-2020-000380.supp1 Supplementary data

All cases and controls provided informed consent to participate.

### Data collection

Study participants were interviewed by trained staff using a structured questionnaire that included information on sociodemographic characteristics (eg, age, sex, residence, ethnicity and occupation), alcohol and maize consumption, as well as use of medications. Study participants also donated blood samples which were used to determine hepatitis B surface antigen (HBsAg), antibody to hepatitis C virus (anti-HCV) and AFB_1_-lysine (AFB_1_-lys) adducts.

### AFB_1_-lysine adduct assessments

The determination of AFB_1_-lys adduct levels was performed by isotope-dilution mass spectrometry^18^ at Dr. John D Groopman’s laboratory at the Johns Hopkins University Bloomberg School of Public Health. Adduct concentrations (pg/AFB_1_-lys/mL serum) were normalised to total serum albumin and expressed as pg AFB_1_-lys adduct/mg albumin. Details of the laboratory methods have been previously described.^3^

### Study covariates

The covariates used in the analysis included age, sex, ethnicity (indigenous vs not indigenous), residence (department of Guatemala and vicinity vs other departments), occupation (farmer vs other), heavy alcohol consumption (alcohol consumption ≥2 drinks for men or ≥1 drink for women per day in the last year, or report of a period in life where five or more drinks every day were consumed), HBsAg and anti-HCV.

### Statistical analysis

Medians and IQRs were calculated for continuous variables, and percentages were used for categorical variables. To examine differences in the characteristics between cases and controls, t-tests or Wilcoxon rank-sum tests were used for continuous variables, and χ^2^ or exact tests were used for categorical variables. Additionally, median and IQR of AFB_1_ for each covariate were computed among the controls, and the differences in the median were assessed by the Wilcoxon rank-sum test. Unconditional logistic regression was used to calculate the ORs and 95% CIs for the association between cirrhosis and the serum AFB_1_-albumin adduct levels by quintiles. A dose–response relationship between cirrhosis and AFB_1_ was examined, and p trends were calculated by scoring (1–5) the quintiles and including the score as a continuous variable in unadjusted and adjusted models. The logistic model selection was based on two different approaches: a stepwise variable selection procedure and examining the change in the estimated ORs by adding covariates, yielding the following covariates for the final model: sex, ethnicity, HBV status, and heavy alcohol consumption. Interaction terms were added to the final model and significance was evaluated using the log rank test. Finally, stratified analysis by sex was performed because of a statistically significant interaction with sex. All statistical analyses were conducted using SAS V.9.4 software, and two-sided p values <0.05 were regarded as statistically significant without adjustment for multiple comparisons.

## Results

Table 1 shows the characteristics of the study participants. The median age of the participants was 55 years (IQR: 48–63). Of the participants, 57% were women. The majority (85%) of individuals resided in the departments of Guatemala and vicinity. Cirrhosis cases were more likely to report heavy alcohol consumption (52%) than were the controls (4%) (p<0.01). The prevalence of HBsAg was low (2.7%) but was statistically higher in cases (7%) than controls (0.5%) (p<0.01). The prevalence of anti-HCV was low (1.3%), and there was no significant difference in prevalence between the cases (3%) and controls (0.5%) (p=0.11). The median AFB_1_ level was significantly higher among the cases (11.4 pg/mg) than among the controls (5.11 pg/mg) (p<0.01).

**Table 1:** Sociodemographic, clinical and other characteristics of individuals by cirrhosis status

Characteristics	Total (N=300)	Cases (n=100)	Controls (n=200)	P value*
Age, median (IQR)	55 (48–63)	54 (47–64)	56 (48–62)	0.07
Sex, n (%)				0.22
Male	129 (43.0)	48 (48.0)	81 (40.5)	
Female	171 (57.0)	52 (52.0)	119 (59.5)	
Indigenous ethnicity, n (%)				0.84
Yes	64 (21.3)	22 (22.0)	42 (21.0)	
No	236 (78.7)	78 (78.0)	158 (79.0)	
Department of residence, n (%)				0.57
Guatemala and vicinity	254 (84.7)	83 (83.0)	171 (85.5)	
Other	46 (15.3)	17 (17.0)	29 (14.5)	
Occupation, n (%)				0.06
Farmer	9 (3.0)	6 (6.0)	3 (1.5)	
Others	291 (97.0)	94 (94.0)	197 (98.5)	
Heavy alcohol consumption, n (%)†				<0.01
Yes	59 (19.8)	51 (52.0)	8 (4.0)	
No	239 (80.2)	47 (48.0)	192 (96.0)	
HBsAg (seropositivity), n (%)†				<0.01
Yes	8 (2.7)	7 (7.0)	1 (0.5)	
No	290 (97.3)	93 (93.0)	197 (99.5)	
Anti-HCV (seropositivity), n (%)				0.11
Yes	4 (1.3)	3 (3.0)	1 (0.5)	
No	296 (98.7)	97 (97.0)	199 (99.5)	
AFB_1_-albumin adduct levels, median (IQR)	7.3 (3.5–14.6)	11.4 (5.7–25.7)	5.11 (2.4–12.0)	<0.01

*P values for categorical variables were obtained from χ^2^ test (sex, indigenous ethnicity, residence and heavy alcohol consumption) or exact test (occupation, HBsAg and HCV status), and for the continuous variables Wilcoxon test (AFB_1_-lysine) or t-test (age).

†Categories do not sum to the total due to missing data.

AFB_1_, aflatoxin B_1_; anti-HCV, antibody to hepatitis C virus; HBsAg, hepatitis B surface antigen; HCV, hepatitis C virus.

Table 2 depicts the median values of AFB_1_-lys adducts by sociodemographic and other characteristics among the controls. Indigenous persons had a significantly higher median AFB_1_-lys adduct levels than did non-indigenous persons (15.2 pg/mg vs 4.8 pg/mg, p<0.01). Similarly, individuals who resided outside the department of Guatemala and vicinity had a significantly higher median AFB_1_ -lys adduct level than did the individuals who lived in the department of Guatemala and vicinity (17.8 pg/mg vs 4.9 pg/mg, p<0.01). No differences in median AFB_1_-lys adduct levels were observed by age, sex, occupation, excessive alcohol consumption, body mass index or HBsAg and anti-HCV status.

**Table 2:** Median and IQR of AFB_1_ by covariates in the control group

Characteristics	AFB_1_-albumin adduct levels* (n=200)	P value†
Age‡		0.45
<56 years	4.7 (2.5–11.7)	
≥56 years	5.5 (2.4–12.3)	
Sex		0.24
Male	6.3 (2.7–12.6)	
Female	4.7 (2.4–11.5)	
Indigenous ethnicity		<0.01
Yes	15.2 (4.3–36.0)	
No	4.8 (2.3–9.5)	
Department of residence		<0.01
Guatemala and vicinity	4.9 (2.4–10.4)	
Other	17.8 (3.4–33.4)	
Occupation		0.95
Farmer	4.6 (1.5–33.4)	
Other	5.2 (2.5–11.9)	
Heavy alcohol consumption		0.20
Yes	4.0 (1.6–6.3)	
No	5.1 (2.5–12.2)	
Body mass index		
<25.0 kg/m^2^	5.5 (3.2–9.5)	0.40
25.0–29.9 kg/m^2^	4.7 (2.3–11.3)	
≥30 kg/m^2^	4.7 (2.3–8.8)	
HBsAg (+)		0.53
Yes	9.5 (9.5–9.5)	
No	5.1 (2.5–11.9)	
Anti-HCV (+)		
Yes	11.5 (11.5–11.5)	0.44
No	5.1 (2.4–12.1)	

*Unit=pg AFB_1_-lysine/mg albumin.

†P values were obtained from Wilcoxon test.

‡The median age among controls is 56.

AFB_1_, aflatoxin B_1_; anti-HCV, antibody to hepatitis C virus; HBsAg, hepatitis B surface antigen.

Table 3 shows the results of the logistic regression analysis for the association between cirrhosis and AFB_1_-lys adduct levels estimated as ORs. Higher levels of AFB_1_ -lys was statistically significant associated with cirrhosis. In the unadjusted analysis, the OR of quintile 5 versus quintile 1 of AFB_1_-lys adduct was 11.55 (95% CI 4.05 to 32.89), while in the adjusted analysis the OR comparing the highest quintile of AFB_1_-lys adduct with the lowest quintile was 12.41 (95% CI 3.23 to 47.74). In both models, there was a significantly increasing trend in the relationship with increasing quintile (p trend=0.001). Using a three-knot restricted linear cubic regression spline, ORs were similar to those of the quintile analysis (data not shown). In addition, adding interaction terms between AFB_1_-lys adducts and the covariates in the final model yielded only one statistically significant interaction, which was between AFB_1_-lys adducts and sex (p=0.01).

**Table 3:** Association of cirrhosis status by quintile of AFB_1_-lysine adduct levels

AFB_1_-albumin adducts	Range (pg/mg albumin)	Cases	Controls	Crude model			Adjusted model*
				OR	95% CI	OR	95% CI
Quintile 1	0.49–2.68	5	54	1.00	–	1.00	–
Quintile 2	2.75–4.98	15	45	3.60	1.21 to 10.67	4.92	1.32 to 18.35
Quintile 3	5.07–9.58	21	39	5.82	2.02 to 16.76	4.85	1.31 to 17.88
Quintile 4	9.66–19.66	27	33	8.84	3.10 to 25.20	12.01	3.34 to 43.14
Quintile 5	19.68–171.58	31	29	11.55	4.05 to 32.89	12.41	3.23 to 47.74
P value for trend					0.001		0.001

Interaction terms were included for the covariates; only AFB_1_ and sex were statistically significant (p=0.01).

*Adjusted for sex, ethnicity, HBV status, and heavy alcohol consumption.

AFB_1_, aflatoxin B_1_; HBV, hepatitis B virus.

The sex-specific analysis of the association between cirrhosis and AFB_1_-lys adducts is presented in table 4. For instance, among women, the adjusted OR comparing the highest quintile of AFB_1_ with the lowest quintile was 5.61 (95% CI 1.24 to 25.38), while among men the equivalent comparison had an adjusted OR of 9.64 (95% CI 1.21 to 76.94).

**Table 4:** Sex-specific association of cirrhosis status by quintile of AFB_1_-lysine adduct levels

AFB_1_-albumin adducts	Range (pg/mg albumin)	Cases	Controls	Crude model		Adjusted model*	
				OR	95% CI	OR	95% CI
Female							
Quintile 1	0.77–2.40	4	30	1.00	–	1.00	–
Quintile 2	2.42–4.36	9	25	2.70	0.74 to 9.82	2.31	0.52 to 10.27
Quintile 3	4.47–7.77	11	24	3.44	0.97 to 12.16	2.02	0.44 to 9.31
Quintile 4	7.83–13.97	12	22	4.09	1.16 to 14.39	3.95	0.96 to 16.35
Quintile 5	14.62–137.42	16	18	6.66	1.93 to 23.07	5.61	1.24 to 25.38
P value for trend					0.002		0.014
Male							
Quintile 1	0.49–3.15	3	22	1.00	–	1.00	–
Quintile 2	3.42–6.59	4	22	1.33	0.27 to 6.67	2.85	0.36 to 22.41
Quintile 3	6.76–12.20	12	14	6.29	1.50 to 26.31	24.85	3.10 to 199.00
Quintile 4	12.49–29.60	16	10	11.73	2.77 to 49.62	25.44	3.26 to 198.64
Quintile 5	29.98–171.58	12	13	6.77	1.61 to 28.54	9.64	1.21 to 76.94
P value for trend					<0.001		0.010

*Adjusted for ethnicity, HBV status, and heavy alcohol consumption.

AFB_1_, aflatoxin B_1_; HBV, hepatitis B virus.

## Discussion

In the current study, cases had significantly higher levels of AFB_1_-lys adducts than did the controls. In addition, there was a statistically significantly increasing trend in the association (OR) between AFB_1_-lys adduct levels and cirrhosis that remained after adjustment for sex, ethnicity, HBV status and heavy alcohol consumption. In addition, evidence of effect modification by sex was observed, with the association between AFB_1_-lys adduct levels and cirrhosis being more pronounced among men than women.

The results of the current study are consistent with other studies from Africa and Asia, where AFB_1_ exposure has historically been high. In The Gambia, a study found that probable exposure to AFB_1_ significantly increased the risk of cirrhosis and that HBV infection had a synergistic effect on the AFB_1_–cirrhosis association.^19^ Similarly, a study in Egypt reported a significantly higher proportion of AFB_1_ signature mutation in *TP53* among persons with chronic liver disease compared with controls.^20^ A Turkish study also reported a significantly higher mean level of AFB_1_ among individuals with cirrhosis compared with controls.^13^ Similarly, a study in Taiwan found that high serum AFB_1_ levels were associated with advanced liver disease.^14^ In addition, a recent nested case–control study in Taiwan reported a dose–response association between AFB_1_-albumin adduct levels and cirrhosis.^15^

Fewer studies have been reported from the Americas, and the results have not been consistent. A study in Mexico found that persons with cirrhosis had high urinary levels of AFB_1_ adducts.^21^ In Brazil, an autopsy study found an association between AFB_1_ residues and chronic liver diseases, including cirrhosis.^22^ In contrast, a US study reported that the AFB_1_ signature mutation in *TP53* was not evident in the tissue of individuals with cirrhosis.^23^

In the current study, the AFB_1_ biomarker used reflects the formation of mutagenic AFB_1_-DNA adducts, and the risk of liver carcinogenesis has been demonstrated to increase with the level of aflatoxin exposure.^24^ A mechanism underlying the possible development of cirrhosis induced by AFB_1_ is not clear. In animal studies, parenchymal changes in the liver caused by steatosis, such as liver cell damage, mononuclear cell infiltration and fibrosis, have been observed after administration of AFB_1_.^25–31^ Furthermore, a recent study has suggested that myofibroblast-like cells may be involved in fibrosis due to AFB_1_ exposure.^31^ Other studies have postulated similar mechanisms, including formation of DNA adducts, protein adducts, and lipid peroxidation.^32^ In addition, it has been suggested that AFB_1_ may act both as a procarcinogen to induce DNA damage and as a liver-damaging agent.^15^ Liver injury has also been shown in experimental animal studies to increase cytochrome p450 enzyme activity, which increases the activation of AFB_1_ and results in greater injury to the liver.^33–35^

Sex difference in the prevalence of cirrhosis has been described in several studies. For example, a US population-based survey reported that cirrhosis was nearly seven times more common among men than women.^36^ The study also reported that 54% of the cases with cirrhosis were attributable to viral hepatitis, excessive alcohol consumption and diabetes,^36^ all of which have been reported to be more common in men than in women.^37–39^ In general, the prevalence and severity of NAFLD also appear to be higher in men compared with women.^40^ Sex differences in AFB_1_ levels and in the metabolism of AFB_1_ have also been observed in some studies. Our previous work in Guatemala found that men had significantly higher circulating levels of AFB_1_-lys adducts than women.^3^ Animal studies have shown that castration of male rats reduced the hepatic metabolism of AFB_1_ (approximately 50%),^41^ and have reported that male rats are more likely to develop AFB-induced glutathione-S-transferase-P-positive hepatocytes (a marker of preneoplastic foci) than do female rats.^42^ This evidence may help to explain the current finding of the AFB_1_–cirrhosis association being more pronounced among men than women.

To our knowledge, this is the first study to assess the association between AFB_1_ and cirrhosis in Guatemala, a population with low prevalence of viral chronic hepatitis and a low rate of heavy alcohol consumption. The strengths of the current study include the use of a robust biomarker of AFB_1_ exposure and the use of a community-based control group that is representative of the underlying general population. In addition, the diagnoses of cirrhosis were determined by ultrasound. Although ultrasound is not the gold standard for diagnosing cirrhosis, it has been reported that the diagnostic accuracy of ultrasound in the detection of cirrhosis is clinically acceptable,^43^ with a sensitivity of 52%–69% and a specificity of 74%–89%.^44^

Limitations of the current study include that the AFB_1_-lys biomarker levels were determined at a single point in time, which may not accurately reflect the cumulative AFB_1_ exposure over time. However, as maize is the most important staple in the Guatemalan diet, it is unlikely that dietary exposure varied greatly over time. In addition, lack of information on other factors among the cases, such as body size and clinical parameters, precluded the ability to examine their effects on the AFB_1_–cirrhosis relationship. As there was no significant relationship between body size and AFB_1_ among the controls, however, it is unlikely that body size would have an effect on the AFB_1_–cirrhosis relationship.

In conclusion, the current study found that cirrhosis was associated with AFB_1_ in Guatemala, a country with a high burden of liver disease. Interventions to mitigate exposure to AFB_1_ as well as efforts to understand the role of other risk factors for cirrhosis may be important to reduce the burden of the disease in Guatemala.

## References

[1] Wogan GN Aflatoxins as risk factors for hepatocellular carcinoma in humans. Cancer Res 1992;52:2114s–8. 1311989

[2] Ferlay JEM, Lam F, Colombet M, et al Global cancer Observatory: cancer today 2018.

[3] Smith JW, Kroker-Lobos MF, Lazo M, et al Aflatoxin and viral hepatitis exposures in Guatemala: molecular biomarkers reveal a unique profile of risk factors in a region of high liver cancer incidence. PLoS One 2017;12:e0189255. 10.1371/journal.pone.0189255 29236788 PMC5728519

[4] Mittal S, El-Serag HB Epidemiology of hepatocellular carcinoma: consider the population. J Clin Gastroenterol 2013;47 Suppl:S2–6. 10.1097/MCG.0b013e3182872f29 23632345 PMC3683119

[5] Asrani SK, Devarbhavi H, Eaton J, et al Burden of liver diseases in the world. J Hepatol 2019;70:151–71. 10.1016/j.jhep.2018.09.014 30266282

[6] Institute for Health Metrics and Evaluation -Guatemala country profile, 2019 Available: http://www.healthdata.org/guatemala [Accessed 2 Sep 2019].

[7] Mokdad AA, Lopez AD, Shahraz S, et al Liver cirrhosis mortality in 187 countries between 1980 and 2010: a systematic analysis. BMC Med 2014;12:145. 10.1186/s12916-014-0145-y 25242656 PMC4169640

[8] Schuppan D, Afdhal NH, cirrhosis L Liver cirrhosis. Lancet 2008;371:838–51. 10.1016/S0140-6736(08)60383-9 18328931 PMC2271178

[9] Suk KT, Kim DJ Staging of liver fibrosis or cirrhosis: the role of hepatic venous pressure gradient measurement. World J Hepatol 2015;7:607–15. 10.4254/wjh.v7.i3.607 25848485 PMC4381184

[10] Stasi C, Silvestri C, Voller F, et al Epidemiology of liver cirrhosis. J Clin Exp Hepatol 2015;5:272. 10.1016/j.jceh.2015.06.002 26628848 PMC4632097

[11] Santos O, Londoño M, Marín J, et al An experience of liver transplantation in Latin America: a medical center in Colombia. Colomb Med 2015;46:8–13. 10.25100/cm.v46i1.1400 26019379 PMC4437281

[12] Wong MCS, Huang J The growing burden of liver cirrhosis: implications for preventive measures. Hepatol Int 2018;12:201–3. 10.1007/s12072-018-9865-y 29679258

[13] Aydın M, Aydın S, Bacanlı M, et al Aflatoxin levels in chronic hepatitis B patients with cirrhosis or hepatocellular carcinoma in Balıkesir, Turkey. J Viral Hepat 2015;22:926–35. 10.1111/jvh.12410 25894298

[14] Chen C-H, Wang M-H, Wang J-H, et al Aflatoxin exposure and hepatitis C virus in advanced liver disease in a hepatitis C virus endemic area in Taiwan. Am J Trop Med Hyg 2007;77:747–52. 10.4269/ajtmh.2007.77.747 17978082

[15] Chu Y-J, Yang H-I, Wu H-C, et al Aflatoxin B_1_ exposure increases the risk of cirrhosis and hepatocellular carcinoma in chronic hepatitis B virus carriers. Int J Cancer 2017;141:711–20. 10.1002/ijc.30782 28509392 PMC5513813

[16] Kroker-Lobos MF, Alvarez CS, Rivera-Andrade A, et al Association between aflatoxin-albumin adduct levels and tortilla consumption in Guatemalan adults. Toxicol Rep 2019;6:465–71. 10.1016/j.toxrep.2019.05.009 31193789 PMC6541741

[17] Torres O, Matute J, Gelineau-van Waes J, et al Human health implications from co-exposure to aflatoxins and fumonisins in maize-based foods in Latin America: Guatemala as a case study. World Mycotoxin J 2015;8:143–59. 10.3920/WMJ2014.1736

[18] McCoy LF, Scholl PF, Schleicher RL, et al Analysis of aflatoxin B1-lysine adduct in serum using isotope-dilution liquid chromatography/tandem mass spectrometry. Rapid Commun Mass Spectrom 2005;19:2203–10. 10.1002/rcm.2045 16015671

[19] Kuniholm MH, Lesi OA, Mendy M, et al Aflatoxin exposure and viral hepatitis in the etiology of liver cirrhosis in the Gambia, West Africa. Environ Health Perspect 2008;116:1553–7. 10.1289/ehp.11661 19057710 PMC2592277

[20] Hosny G, Farahat N, Tayel H, et al Ser-249 TP53 and CTNNB1 mutations in circulating free DNA of Egyptian patients with hepatocellular carcinoma versus chronic liver diseases. Cancer Lett 2008;264:201–8. 10.1016/j.canlet.2008.01.031 18313840

[21] Alvarez-Banuelos M, Carvajal-Moreno M, Mendez-Ramirez I, et al Free and DNA adducted aflatoxins in chronic liver diseases that predispose patients to hepatocellular carcinoma in Mexico. J Cancer Sci Ther 2015;7:274–82.

[22] Ramalho LNZ, Porta LD, Rosim RE, et al Aflatoxin B_1_ residues in human livers and their relationship with markers of hepatic carcinogenesis in São Paulo, Brazil. Toxicol Rep 2018;5:777–84. 10.1016/j.toxrep.2018.07.005 30101081 PMC6082919

[23] Jiao J, Niu W, Wang Y, et al Prevalence of Aflatoxin-Associated TP53R249S Mutation in Hepatocellular Carcinoma in Hispanics in South Texas. Cancer Prev Res 2018;11:103–12. 10.1158/1940-6207.CAPR-17-0235-AT PMC5811406 29089331

[24] Wild CP, Miller JD, Groopman JD Mycotoxin control in low- and middle-income countries. Lyon (Fr: International agency for research on cancer, 2015. 27030861

[25] Rosmael J Are aflatoxins a cause of parenchymal liver disease in humans? University of Oslo 2016.

[26] Karaman M, Ozen H, Tuzcu M, et al Pathological, biochemical and haematological investigations on the protective effect of alpha-lipoic acid in experimental aflatoxin toxicosis in chicks. Br Poult Sci 2010;51:132–41. 10.1080/00071660903401839 20390578

[27] Seffner W, Schiller F, Lippold U, et al Experimental induction of liver fibrosis in young guinea pigs by combined application of copper sulphate and aflatoxin B1. Toxicol Lett 1997;92:161–72. 10.1016/S0378-4274(97)00052-0 9334826

[28] Wogan GN Chemical nature and biological effects of the aflatoxins. Bacteriol Rev 1966;30:460–70. 10.1128/MMBR.30.2.460-470.1966 5327461 PMC441006

[29] Ortatatli M, Oğuz H, Hatipoğlu F, et al Evaluation of pathological changes in broilers during chronic aflatoxin (50 and 100 ppb) and clinoptilolite exposure. Res Vet Sci 2005;78:61–8. 10.1016/j.rvsc.2004.06.006 15500841

[30] Wouters ATB, Casagrande RA, Wouters F, et al An outbreak of aflatoxin poisoning in dogs associated with aflatoxin B1-contaminated maize products. J Vet Diagn Invest 2013;25:282–7. 10.1177/1040638713477409 23417078

[31] Arana S, Alves VAF, Sabino M, et al Immunohistochemical evidence for myofibroblast-like cells associated with liver injury induced by aflatoxin B1 in rainbow trout (Oncorhynchus mykiss). J Comp Pathol 2014;150:258–65. 10.1016/j.jcpa.2013.07.003 24016778

[32] Moudgil V, Redhu D, Dhanda S, et al A review of molecular mechanisms in the development of hepatocellular carcinoma by aflatoxin and hepatitis B and C viruses. J Environ Pathol Toxicol Oncol 2013;32:165–75. 10.1615/JEnvironPatholToxicolOncol.2013007166 24099430

[33] Kirby GM, Batist G, Alpert L, et al Overexpression of cytochrome P-450 isoforms involved in aflatoxin B1 bioactivation in human liver with cirrhosis and hepatitis. Toxicol Pathol 1996;24:458–67. 10.1177/019262339602400408 8864187

[34] Kirby GM, Chemin I, Montesano R, et al Induction of specific cytochrome P450s involved in aflatoxin B1 metabolism in hepatitis B virus transgenic mice. Mol Carcinog 1994;11:74–80. 10.1002/mc.2940110204 7916995

[35] Gemechu-Hatewu M, Platt KL, Oesch F, et al Metabolic activation of aflatoxin B1 to aflatoxin B1-8,9-epoxide in woodchucks undergoing chronic active hepatitis. Int J Cancer 1997;73:587–91. 10.1002/(SICI)1097-0215(19971114)73:4&lt;587::AID-IJC21&gt;3.0.CO;2-5 9389576

[36] Scaglione S, Kliethermes S, Cao G, et al The epidemiology of cirrhosis in the United States: a population-based study. J Clin Gastroenterol 2015;49:690–6. 10.1097/MCG.0000000000000208 25291348

[37] Kautzky-Willer A, Harreiter J, Pacini G Sex and gender differences in risk, pathophysiology and complications of type 2 diabetes mellitus. Endocr Rev 2016;37:278–316. 10.1210/er.2015-1137 27159875 PMC4890267

[38] Nolen-Hoeksema S Gender differences in risk factors and consequences for alcohol use and problems. Clin Psychol Rev 2004;24:981–1010. 10.1016/j.cpr.2004.08.003 15533281

[39] Wu EM, Wong LL, Hernandez BY, et al Gender differences in hepatocellular cancer: disparities in nonalcoholic fatty liver disease/steatohepatitis and liver transplantation. Hepatoma Res 2018;4. 10.20517/2394-5079.2018.87. [Epub ahead of print: 18 Oct 2018]. PMC6347119 30687780

[40] Lonardo A, Nascimbeni F, Ballestri S, et al Sex differences in nonalcoholic fatty liver disease: state of the art and identification of research gaps. Hepatology 2019;70:1457–69. 10.1002/hep.30626 30924946 PMC6766425

[41] Gurtoo HL, Motycka L Effect of sex difference on the in vitro and in vivo metabolism of aflatoxin B1 by the rat. Cancer Res 1976;36:4663–71. 1000509

[42] Tsuji K, Gopalan P, Lehmann K, et al Species and sex differences of aflatoxin B1-induced glutathione S-transferase placental form in single hepatocytes. Cancer Lett 1992;66:249–54. 10.1016/0304-3835(92)90254-S 1451106

[43] Procopet B, Berzigotti A Diagnosis of cirrhosis and portal hypertension: imaging, non-invasive markers of fibrosis and liver biopsy. Gastroenterol Rep 2017;5:79–89. 10.1093/gastro/gox012 PMC5421457 28533906

[44] Huber A, Ebner L, Heverhagen JT, et al State-Of-The-Art imaging of liver fibrosis and cirrhosis: a comprehensive review of current applications and future perspectives. Eur J Radiol Open 2015;2:90–100. 10.1016/j.ejro.2015.05.002 26937441 PMC4750581

