# Supplementary material for: Aflatoxin B1 exposure and liver cirrhosis in Guatemala: a case–control study
Source: BMJ Open Gastroenterol. 2020 Jul 7;7(1):e000380. doi: 10.1136/bmjgast-2020-000380 (PMC7342465; doi:10.1136/bmjgast-2020-000380)

Supplemental materials

Figure S1. Flow chart of inclusion and exclusion criteria for the cases and controls

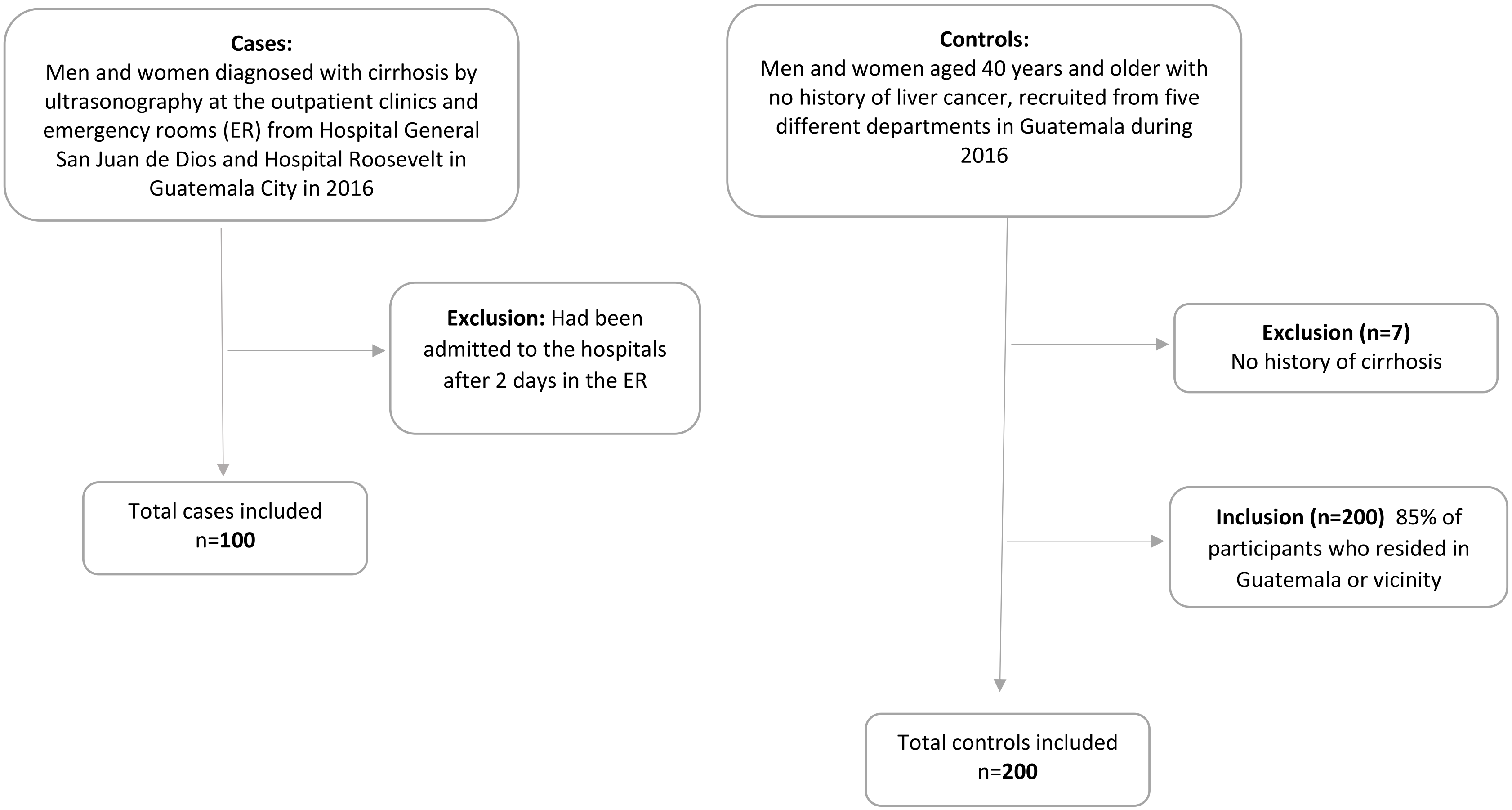

Supplement: Supplementary data [file bmjgast-2020-000380supp001.pdf]
